# Studying patients with autoinflammatory diseases: the past, present, and a perspective for the future

**DOI:** 10.1186/1546-0096-13-S1-P187

**Published:** 2015-09-28

**Authors:** JS Hausmann, C Biggs, D Goldsmith, F Dedeoglu

**Affiliations:** 1 Boston Children's Hospital, Rheumatology, Boston, MA, USA; 2 Beth Israel Deaconess Medical Center, Rheumatology, Boston, MA, USA; 3 St. Christopher's Hospital for Children, Drexel University College of Medicine, Rheumatology, Philadelphia, PA, USA

## Introduction

Autoinflammatory diseases (AID) are rare disorders characterized by recurrent episodes of systemic and organ-specific inflammation. Studying AID has been limited by the difficulty in finding and enrolling large numbers of patients with these rare illnesses. We used a traditional retrospective chart review to describe patients with AID at a single academic medical center, and compared the results with those of participants within the Childhood Arthritis and Rheumatology Research Alliance (CARRA) Registry, a multicenter observational pediatric rheumatic disease registry in North America. We discuss the benefits and limitations of these types of studies, and suggest new ways to conduct future research.

## Methods

We included patients with a clinical or genetic diagnosis of AID. We conducted a retrospective chart review at Boston Children's Hospital (BCH) from 2002-2012. Charts were identified by keywords and billing codes related to AID, and patients with CRMO were excluded. We also conducted a cross-sectional study of children with AID enrolled in the CARRA registry from May 2011 to December 2013.

## Results

At BCH, we identified 169 subjects with AID. In the CARRA registry, of 9,523 subjects enrolled, 87 patients had AID. The distribution of diagnoses from patients at BCH included PFAPA (48%), FMF (22%), TRAPS (2%), MKD (2%), NOMID (2%), FCAS (2%), and undefined (23%). In the CARRA registry, diagnoses included CRMO (39%), FMF (17%), PFAPA (10%), TRAPS (7%), NOMID (3%), MWS (3%), SAPHO (2%), PAPA (1%), FCAS (1%), MKD (1%), and undefined (14%).

## Conclusions

### The past

Using the traditional method of a single-center retrospective chart review, we described the patients with AID seen at BCH over a 10-year period. This research required little cost and relatively little time. Limitations included incomplete documentation of some patients, and the variety of AID which were identified.

### The present

The CARRA registry was a multicenter effort where patients were enrolled at a faster rate, and with a greater variety of diagnoses. However, this registry required significant financial investments in technology and operational costs. PFAPA, the most common pediatric AID, represented only a minority of subjects within the CARRA registry, suggesting an enrolment bias, perhaps due to the time required for consent, enrolment, and data-uploading process.

### The future

Future registries will likely be integrated into the patient's electronic health records, avoiding many of the current barriers to research. In addition, we believe that online patient communities can contribute valuable information regarding patient-reported outcomes. In future studies, we plan to empower and engage patients with AID through social media to collaborate in design and research implementation. Our efforts will exponentially expand the number of patients available to participate in research, and will provide more complete and meaningful data.

